# A health education outreach partnership between an academic medical library and public library: lessons learned before and during a pandemic

**DOI:** 10.5195/jmla.2022.1413

**Published:** 2022-04-01

**Authors:** Stephanie M. Swanberg, Nancy Bulgarelli, Mithya Jayakumar, Erin Look, Tyler B. Shubitowski, Rose Wedemeyer, Emily W. Yuen, Victoria C. Lucia

**Affiliations:** 1 sswanberg@msp.edu, User Services Librarian, Moustakas Johnson Library, Michigan School of Psychology, Farmington Hills, MI. Previously, Associate Professor, Information Literacy & eLearning Librarian, Medical Library, Oakland University William Beaumont School of Medicine, Rochester, MI (start of study); 2 bulgarel@oakland.edu, Associate Professor, Oakland University Libraries, Rochester, MI. Previously, Associate Professor and Director, Medical Library, Oakland University William Beaumont School of Medicine, Rochester, MI (start of study); 3 msjayakumar@oakland.edu, Medical Student, Oakland University William Beaumont School of Medicine, Rochester, MI; 4 looke@ahplibrary.org, Youth Services Coordinator, Auburn Hills Public Library, Auburn Hills, MI; 5 tshubitowski@oakland.edu, Medical Student, Oakland University William Beaumont School of Medicine, Rochester, MI; 6 ragutier@oakland.edu, Director of Education Training, Oakland University William Beaumont School of Medicine, Rochester, MI; 7 eyuen@oakland.edu, Medical Student, Oakland University William Beaumont School of Medicine, Rochester, MI; 8 lucia@oakland.edu, Associate Professor, Department of Foundational Medical Studies, Oakland University William Beaumont School of Medicine, Rochester, MI

**Keywords:** outreach, community engagement, health education, health literacy, public library, academic medical library

## Abstract

**Background:**

Public libraries serve as community centers for accessing free, trustworthy health information. As such, they provide an ideal setting to teach the local community about health and health literacy, particularly during public health crises like the COVID-19 pandemic. Since 2018, an outreach partnership between an academic medical library and public library has developed, delivered, and continuously evaluated a health education program targeting public library users.

**Case Presentation:**

Health education activities were integrated into three existing public library programs: adult workshops, child and family programming, and circulating family activity kits. Prior to COVID-19, events were held at the public library, which then pivoted online during the pandemic. An interprofessional team approach combined the expertise of academic medical and public librarians, medical school faculty and staff, and medical students in developing the educational programs. Twelve in-person and five virtual programs were offered, and five circulating health education family kits were launched. Activities were assessed using program evaluation surveys of the adult and children's programs and circulation statistics of the kits.

**Conclusions:**

This case report showcases the lessons learned from implementing a longitudinal outreach partnership between an academic medical library and public library before and during the COVID-19 pandemic. The interprofessional team approach and flexibility in program design and delivery in both the in-person and virtual environments proved critical to the success of the partnership. This partnership could serve as a model for other libraries interested in pursuing interprofessional collaborations in educating local communities on healthy behavior and health information–seeking practices.

## BACKGROUND

Outreach to the local community raises awareness of free, authoritative resources and promotes health and health information literacy [1]. The importance of outreach has become more pressing during the COVID-19 pandemic, during which an overwhelming amount of information has made it difficult for the public to differentiate between reliable information and misinformation [2, 3]. A recent study revealed that although 87.6% of consumers rated government websites as the most trusted source of pandemic information, over 90% still relied on traditional sources like TV, radio, and newspapers, and trust in government sources decreased as the pandemic progressed [4]. Additionally, choice of sources was often driven by sociodemographic characteristics, such as age, gender, education level, and political beliefs [4].

Community members often turn to their local library as a trusted source of health information [5–7]. Public libraries are well positioned to address this need through programming, collections, and reference services. COVID-19 has drawn increased attention to the essential role of public libraries in health information literacy [2, 8–10]. However, studies have found that public librarians sometimes struggle to answer health information–related requests due to lack of knowledge and training [6, 7, 11–13]. Partnerships between academic medical libraries and public libraries provide opportunities to share expertise and resources in addressing consumer health information needs, such as medical librarians conducting training for public library staff and offering collaborative health programming [14–18].

This case study reports on the development, implementation, and evaluation of a health education partnership between the Oakland University William Beaumont School of Medicine (OUWB) Medical Library and Auburn Hills Public Library (AHPL). It represents an interprofessional collaboration between academic medical librarians, public librarians, medical school faculty and staff, and medical students to educate the local community on positive health behaviors and health information–seeking practices.

## CASE PRESENTATION

Community outreach and engagement are integral to OUWB's mission, vision, and values and are a priority for the medical school and university [19–21]. AHPL is located near the university and serves a diverse community including adults over sixty-five, children, and over 5,000 families [22]. In addition, a greater percentage of Auburn Hills residents are uninsured and have higher unemployment rates than Michigan state averages [23]. Integrating health information activities into existing library programs provides an effective means of reaching many members of the community in teaching them how to access, evaluate, and use health information.

The collaboration began in August 2018 when the OUWB Medical Library approached AHPL about their interest in and patron need for health education and health information literacy. Both libraries shared a mutual interest in increasing health programming, promoting healthy behaviors, and raising awareness of free, reliable health information resources. As a pilot, an adult workshop and two children's programs were offered at the public library in fall 2018. Based on feedback from public librarians and attendance, the pilot was deemed successful. This subsequently evolved into a partnership focused on integrating health education into three existing public library programs: adult workshops, child and family programming, and circulating family activity kits, all of which pivoted online during the COVID-19 pandemic. In addition, after the pilot year, the team received a National Network of Libraries of Medicine All of Us Public Library Partnership Award to fund supplies and develop and evaluate additional programs. This funding resulted from a National Institutes of Health/National Library of Medicine partnership to promote the *All of Us* Research Program and support increasing health literacy activities at public libraries [24]. The Oakland University institutional review board determined this project was non-human subjects research.

### Adult workshops

#### Description

AHPL hosts regular weekend workshops targeting adults on a variety of topics, with a special emphasis on developing programming for seniors. The adult services librarian and the medical librarian collaborated to add an expert speaker series on popular health topics; three in-person sessions were delivered at the public library on influenza and hepatitis A, pain management, and fad diets. For each session, the medical librarian invited a medical school faculty member to develop and coteach a workshop on the topic, while the public librarian promoted and hosted the event.

Pre-COVID-19, sessions were one hour and included the expert speaker presentation, a brief demonstration of related online health resources by a medical librarian, a question-and-answer portion, and a post-session evaluation. During the pandemic, sessions pivoted to mini virtual asynchronous offerings. An OUWB faculty member demonstrated mindfulness practices via a twenty-minute “follow-along” recording embedded on the AHPL website. Table 1 reports the total number of attendees and views for each session.

**Table 1:** Health education programs summary, 2018–2021

Year	Activity name	Activity format	Attendees / views / circulations	Learning objectives
Adult workshops				
2018–2019	Everything You Need to Know about the Flu & Hepatitis A	in-person	3	Identify myths and facts related to flu and hepatitis A and the benefits of vaccines Describe methods of preventing the spread of flu and hepatitis A Identify freely available online resources for good health information
2018–2019	Uncovering Recent Trends in Medical Pain Management	in-person	5	Define opioids and medical marijuana Explain the effects of opioids and medical marijuana on the body Describe proper drug storage and disposal methods Identify freely available online resources for drug information
2019–2020	The Ins & Outs of Popular Diets & Their Impact on Your Health	in-person	7	Identify the types of fad diets commonly used by the general public Discuss the potential benefits and risks of fad diets Identify freely available online resources for diet and nutrition information
2020–2021	Mini-Relaxation Techniques	recording	40	Define breathing, grounding, and visualization mindfulness techniques Practice and reflect on three mindfulness techniques
Total			15 attendees; 40 views	
Children & family programs				
2018–2019	Germs & Handwashing (offered 3 times)	in-person	20	Explain the importance of proper handwashing to prevent sickness Demonstrate proper handwashing to prevent the spread of germs Demonstrate skills to reduce the spread of germs
2018–2019	Eat the Rainbow	in-person	8	Explain the importance of eating a variety of foods from all of the food groups Classify food into food groups Describe characteristics of foods and beverages that should be limited Identify examples of food that could be eaten as a healthy snack
2018–2019	Your Amazing Body	in-person	15	Identify the location of various organs in the human body Explain the importance of the digestive, respiratory, and circulatory systems to our bodies Simulate how the digestive, respiratory, and circulatory systems work
2018–2019	Exercise	in-person	7	Explain the importance of regular physical activity in keeping the body healthy Describe how different exercises impact various parts of the body (e.g., heart, muscles, brain) Practice exercise techniques Create an exercise plan for a one-week period
2019–2020	Sun Safety	in-person	12	Explain the importance of protecting your skin from the sun while outdoors Identify items that can protect your skin from the sun Simulate how the sun can harm skin cells and the potential long-term effects of this
2019–2020	Spooky Bones	in-person	12	Explore the functions of the skeletal system and how our bones change as we grow Demonstrate activities that strengthen and keep our bones healthy Identify the places in our bodies where bones connect
2019–2020	Healthy Hearts	in-person	7	Explore the functions of the heart Learn about healthy heart sounds Learn about how the heart pumps blood throughout our body
2020–2021	Digestive Tract	take-home kit & follow-along recording	15	Explain the importance of the digestive system in our bodies Show how the digestive system works using Play-Doh
2020–2021	Skeletal System	take-home kit & follow-along recording	10	Explain the importance of the skeletal system to our bodies Identify the major bones in the body Create a basic model of the skeletal system
2020–2021	Safety and Wellness during COVID-19	livestreamed and recorded	87	Discuss best practices for family safety and wellness during the COVID-19 pandemic Share COVID-19 concerns and questions
2020–2021	Mental Wellness during COVID-19	livestreamed and recorded	Unknown—technical error in session recording	Identify trends in mental wellness during COVID-19 Articulate strategies for addressing mental health concerns Identify resources for getting help for mental health concerns Share COVID-19 concerns and questions
Total			81 attendees; 112 views	
Circulating health kits				
2019–2020	Germs & Handwashing	circulating kit	6	Explain the importance of proper handwashing to prevent sickness Demonstrate proper handwashing to prevent the spread of germs Demonstrate skills to reduce the spread of germs
2019–2020	Nutrition	circulating kit	6	Classify food into food groups Identify foods and beverages that are healthy and those that should be limited
2019–2020	Exercise	circulating kit	7	Explain the importance of regular physical activity in keeping the body healthy Practice exercise techniques
2019–2020	Mindfulness	circulating kit	6	Identify different types of mindfulness practices, including yoga and breathing Practice mindfulness exercises
2019–2020	Medical Careers	circulating kit	11	Explain how doctors, nurses, and veterinarians care for people and animals Practice care skills including taking a temperature, listening to a heart, and applying a bandage
Total			36 circulations	

#### Evaluation

After the pilot, an anonymous survey was developed to evaluate general library programming and that day's workshop [25]. Nine of 12 participants from the two subsequent workshops completed the survey, a response rate of 75%. All respondents (n=9) rated the workshops as good or excellent (n=9). They reported learning about the event through library social media (n=4), on-site library advertising (n=3), or the library website, library newsletter, local newspaper, or word of mouth (n=1, each). Recommendations for future workshop topics included physical activity, Alzheimer's disease, addiction, and cannabidiol.

### Children & family programs

#### Description

AHPL offers weekly family story times (for preschool-aged children) and monthly science, technology, engineering, arts, and math (STEAM) programming (for elementary school–aged children). The youth services librarian suggested integrating health education into these two popular existing programs and brainstormed topic ideas with the medical librarian. The medical school librarian, faculty, and staff then partnered with the OUWB Lifestyle Medicine Student Interest Group to develop and deliver the children's programming, with the public librarian managing marketing and hosting the event. From 2018 to 2020, nine in-person children's events were hosted on the following topics: germs and handwashing, nutrition, exercise, skincare, and various body systems including cardiovascular, respiratory, digestive, and skeletal (Table 1).

Pre-COVID-19, each program featured the youth services librarian reading a book on the health topic followed by hands-on activities led by medical students. Eighty-one children attended these programs over three years while over twenty OUWB student volunteers were engaged. With the onset of the pandemic, these sessions were converted to instructional videos and a take-home activity kit. Instructions directed children to watch an online video in which a medical student read a book on the designated topic and led them through an activity completed with the supplies provided. These were created as single-use kits that families kept. To date, two take-home activities have been created on the digestive tract and the skeletal system with fifty kits distributed to families.

To ensure the community had access to reputable health information during the pandemic, AHPL offered a new, virtual health education program: Ask-a-Doc. During this program, a public librarian interviewed a physician about COVID-19 and gave families the opportunity to ask questions. The medical librarian recruited clinical faculty and the public librarian developed interview questions, advertised, and hosted the event. Two sessions were offered: “Safety and Wellness during COVID-19” and “Mental Wellness during COVID-19.” Each session was thirty to sixty minutes long and held virtually via Zoom while live-streamed to the public library's Facebook page. The recordings have over eighty views to date.

#### Evaluation

In the second year of the partnership, two evaluation tools were implemented to assess these programs: a post-session evaluation survey for child participants and a medical student communication observation tool to evaluate their interactions.

**Figure 1:**
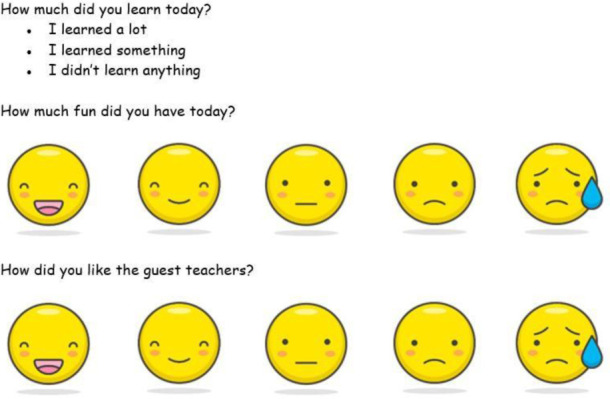
Children's program evaluation survey

As the ages of the child participants ranged from five to twelve years, the guided survey tool needed to be brief and simple to accommodate all ages. A three-question survey was developed with two questions using an emotion scale of smiley faces as reported by previous studies [26, 27] (Figure 1). All but one child participant completed the program survey (n=30) over the three programs offered during the second year. Most children selected “I learned a lot” (n=24), with a majority selecting either the super smiley or smiley face when asked how much fun they had (n=28) and how they liked the medical student teachers (n=28).

Image icon credits: All were designed by Vincent Le Moign under a CC BY 4.0 license and available via Wikimedia Commons <https://commons.wikimedia.org/wiki/File:006-grinning-face-with-smiling-eyes.svg>

In addition, the Teach-back Observation Tool, typically used to observe health provider communication skills with patients, was modified for a health education setting to provide feedback on medical student teaching performance [25, 28]. The tool assesses communication items using a dichotomous scale (yes/no) and a comments section to identify characteristics such as caring tone, welcoming body language, limited use of medical jargon, and asking open-ended questions. At each session, two to three observers (medical school faculty and medical librarians) completed the observation checklist for the medical student facilitators. The collected observation data indicated that students consistently displayed a caring tone and welcoming body language and often asked open-ended questions. They maintained professionalism while entertaining the children and creating a fun, engaging environment. Areas for improvement were (1) avoiding medical jargon and adjusting language to the appropriate literacy level and (2) making sure learners grasped a topic, particularly when introducing an activity. However, as many students volunteered at several sessions, observers commented on improvements in communication skills over time. Students were able to use skills and techniques learned from their clinical training curriculum, such as engaging patients and distilling complex information, and adjust them to a younger age group. In addition, informal feedback was gathered from medical students during session debriefs, in which they indicated that participating in these activities contributed to their ability to work with diverse populations and ages.

### Circulating health kits

In addition to offering educational programs, AHPL creates circulating activity backpacks as part of their children's collection. Each backpack is themed and includes books, an activity sheet, online resource links, and educational toys or games. A medical student, youth services librarian, and medical librarian selected five topics for health-related kits based on the perceived interest of families: germs and handwashing, nutrition, exercise, mindfulness, and medical careers. Five kits were developed and released into circulation in February 2020, but paused due to COVID. Kits began recirculating in July 2021 with thirty-six total checkouts to date (Table 1).

## DISCUSSION

### Lessons learned: successes

Previous studies of academic medical and public library partnerships fall into two main approaches: (1) medical librarians training public library staff in health information resources [14, 15] and (2) collaborating to plan and offer health programming targeting library patrons [16–18]. Many attributes of successful library outreach previously reported in the literature were found in this project, including sharing mutual interests and goals, aligning with institutional missions, and addressing community interests and needs [1, 20, 29, 30]. This project also revealed unique elements that could be adapted by other libraries seeking to implement similar programs. These include utilizing an interprofessional team approach beyond librarianship and implementing flexible delivery methods as needed, in this case by shifting online during COVID-19 to address emerging health concerns of the community.

#### Interdisciplinary team approach

Collaboration, both within and outside librarianship, proved invaluable to the success of this outreach partnership. Without the interprofessional collaboration between the medical library, public library, and medical school, we would not have been able to successfully develop and deliver as many programs and events. First, the public librarians had a solid knowledge of patron interests and popular circulating item topics to guide the team; this provided the opportunity for directed health topics to be addressed. Also, having an established audience who frequently attended existing library programs allowed us to leverage existing opportunities to introduce the community to these health education activities. Second, the participation of medical school faculty and staff provided the team with additional resources and supplies. Because these medical school faculty and staff had previously developed programming with local public school districts, they also brought expertise in K-12 education and community engagement to the team. Also, there was sharing of a robust set of existing and newly acquired supplies, educational materials, and educational activities that could be modified for specific age groups. Finally, collaborating with leaders from a medical student interest group also allowed for efficient recruitment of engaged volunteers to facilitate events at the public library. Many volunteers also had previous experience working with K-12 students and were able to develop experiential sessions for the children and family programming. See Table 2 for a summary of roles and responsibilities.

**Table 2:** Summary of interprofessional team roles & responsibilities

Team member	Number involved	Responsibilities
Academic medical librarians	2	served as primary coordinator and communication channel for the interprofessional team coordinated all activity planning with other team members: brainstormed topics, activities, lesson plans, and evaluation methods organized and purchased supplies organized volunteer training sessions managed event logistics of set-up and takedown led funding applications
Public librarians	3	provided insight on patron interest and circulation statistics brainstormed topics and reviewed lesson plans for appropriateness handled all event marketing and promotion served as hosts for all programs by: providing spaces and technology, both physical and virtual, for hosting programs introducing speakers or volunteers leading story time portions of activities and participating in experiential activities
Medical school faculty & staff	2	provided expertise in K-12 education and leaders in organizing other medical school community engagement activities provided guidance and forms for establishing the formal affiliation agreement between the medical library and public library brainstormed topics, activities, lesson plans, and evaluation methods shared supplies provided initial introduction to medical student organizations served as co-investigators and provided expertise on funding applications
Medical student interest group leaders	3	applied past experiences in K-12 education and public health developed and/or refined lesson plans and kits served as facilitators for children's programs recruited and trained medical student volunteers
Medical student volunteers	5–6 per event	served as facilitators for children's programs received credit for community service course requirement if needed
Guest expert speakers	1 per event	medical school faculty and physicians outside of the planning team invited to teach workshops both in person and virtually

#### Flexible delivery method

Mode of delivery requires a flexible approach and basic understanding of various accessible online delivery platforms. Pre-COVID-19, the partnership was steadily growing with all programs hosted in person at the public library. Children's programs were especially well attended, with evaluation data indicating that the children enjoyed and learned from these sessions. As COVID-19 closed both the university and public library, planned in-person sessions were cancelled. One success was observing and learning from the experiences of the public librarians as they experimented in offering virtual programs through different platforms. Our health education activities resumed by following their lead and integrating into the library's virtual and socially distanced programs. This included developing asynchronous recorded instructional videos and posting them on YouTube, hosting Zoom sessions simultaneously live-streamed to Facebook, and offering take-home kits for families. This approach allowed the health education activities to continue even with the challenges presented by the pandemic.

While integrating in-person events into existing public library programs proved successful in terms of attendance and engagement, pivoting to virtual programs revealed several takeaways for planning education sessions in the post-pandemic future. Virtual sessions were easier to implement without the logistical considerations of volunteer recruitment and training or setup and takedown of physical spaces, which reduced planning and implementation time. Virtual programs also reached a larger audience. For children's activities, fifty families were able to pick up take-home kits, compared to an average of twelve attendees at each in-person session. For adult programs, there was an average of sixty views of live-streamed or asynchronous recorded sessions compared to an average of five participants at adult in-person sessions. This suggests that post-pandemic outreach programming should consider hybrid (live in-person session and accompanying asynchronous recording) or fully virtual options. A hybrid approach where participants can watch at their leisure provides flexibility for interested patrons by accommodating busy schedules. These opportunities mirror those reported in the literature, which anticipate libraries offering a greater number and variety of programs and services in flexible, hybrid formats post-pandemic [8, 10, 31]. However, a major drawback of fully virtual programs was the lack of personal interactions and experiential learning, including (1) engaging with medical students, physicians, and faculty directly, (2) opportunities to ask questions, and (3) having personalized learning experiences. While hosting educational and interactive events accounted for a large portion of this project, take-home kits proved very popular. As such, collection development opportunities can also be maximized post-COVID-19 through building health-related circulating items.

### Lessons learned: challenges

#### Marketing

Special consideration should be given to active versus passive marketing approaches when advertising events not integrated into existing programs. Integrating activities into recurring weekly library children's programs yielded the most success along with marketing the children's programs on the library's social media platforms and print flyers. The adult programs were mostly passively marketed: posted on the website events calendar, print flyers, and in a quarterly print newsletter sent months in advance. As a result, attendance at these events was lower than the children's events. Future marketing efforts should include more active approaches such as utilizing social media, reaching out to local senior centers or communities, and planning events closer to mass media coverage, such as the influenza and hepatitis A workshop that was offered too long after a statewide hepatitis A outbreak.

#### Engaging specific audiences online

Pre-COVID-19, topic selection for adult programs was driven by trends in circulation requests, direct questions from patrons, and current events. Keeping abreast of topics of interest to seniors posed the unique challenge of integrating easy-to-understand online resources while dispelling possible misinformation or myths. During COVID-19 and the subsequent public library closure, the adult services librarian reported that attendance at virtual synchronous adult programs was poor and suggested developing short asynchronous recordings. Mindfulness was selected as the topic of the first recording by the public librarian to help community members manage pandemic-related stress [32]. However, anticipating the health literacy needs and questions of patrons became problematic as the pandemic progressed and the public was flooded with competing and contradictory information from various media and personal sources. In addition, it is unclear if seniors, the target adult audience, felt comfortable with or had adequate digital literacy skills to engage in online programs. However, as a recent Pew Research Center survey found that 25% of Americans aged sixty-five and older do not use the Internet, online programming may unintentionally exclude this age group [33]. As such, post-COVID-19, there may be additional outreach opportunities both online and in person, such as hosting library events directly at senior community centers or housing facilities. In the future, considerations need to be made to ensure that this population is included and if digital literacy may be an opportunity for future education.

#### Enduring turnover & sustainability

Sustainability is one of the most commonly reported challenges of outreach [1, 20, 29, 30]. As institutional missions evolve, interests change, and staff turnover occurs, outreach projects are often the first to be affected. COVID-19 has also impacted the workforce with many organizations experiencing unprecedented rates of staff turnover due to changing work environments and demands [34]. Partnerships such as the one described here may be particularly vulnerable to such changes [30]. During the course of the partnership, the primary coordinating medical librarian left for another position and other staffing changes in the medical library caused a shift to reprioritize basic library services. However, the strong foundations of this partnership and the interdisciplinary team approach contributed to programmatic resiliency, with champions from the medical school currently working to preserve the partnership with the public library.

Public libraries serve as cornerstones of their communities and are trusted sources of free, reliable information, including health information. The COVID-19 pandemic showcased the increased need and importance of health literacy in driving health care decision-making. Libraries can promote their role in addressing this need by offering programs and building collections. This case report demonstrates the ongoing importance of educational outreach initiatives in addressing the changing health landscape and ongoing health information needs of the community, particularly during COVID-19. Partnerships between medical and public libraries, especially those that leverage the expertise of health care professionals, can create successful interprofessional teams that are flexible, sustainable, and capable of sharing valuable resources, time, and both physical and virtual spaces.

## Data Availability

Data associated with this article are available in the OUR@Oakland institutional repository at <http://hdl.handle.net/10323/11355>. Education materials developed from this project, including PowerPoints, lesson plans, and supply lists, are available from the corresponding author by request.
